# Supplementary material for: Model-based decoupling of evoked and spontaneous neural activity in calcium imaging data
Source: PLoS Comput Biol. 2020 Nov 30;16(11):e1008330. doi: 10.1371/journal.pcbi.1008330 (PMC7728401; doi:10.1371/journal.pcbi.1008330)
Supplement: S1 Table — Parameters related to time are defined with respect to imaging rate. Listed values of π and σ2 are defaults, but are varied over the range specified in parentheses. (PDF) [file pcbi.1008330.s003.pdf]

Parameter	Interpretation	Value
N	Number of neurons	200
T	Simulation duration (frames)	2000
–	Imaging rate (Hz)	2.1646
τ_r	Calcium rise time constant (frames)	2.6
τ_d	Calcium decay time constant (frames)	5.3
K	Number of stimuli	10
–	Stimulus repetitions	5
–	Interstimulus interval (frames)	39
L	Number of latent factors	4
ξ	Probability of latent factor event (per frame)	0.1
π	Probability of private spontaneous event (per frame)	0.05 $(0.0$ - $0.3)$
γ_x	Mean intensity of latent event	0.1
γ_z	Mean intensity of private spontaneous event	0.1
σ^2	Imaging noise variance	0.16 $(0.04$ - $0.4)$
