# Supplementary material for: Model-based decoupling of evoked and spontaneous neural activity in calcium imaging data
Source: PLoS Comput Biol. 2020 Nov 30;16(11):e1008330. doi: 10.1371/journal.pcbi.1008330 (PMC7728401; doi:10.1371/journal.pcbi.1008330)
Supplement: S3 Table — Zebrafish 5 corresponds to the example used in Figs 1–4 and S1 and S4–S7 Figs. (PDF) [file pcbi.1008330.s005.pdf]

	Zebrafish							
	1	2	3	4	5	6	7	8
Correlation coefficient mean	0.67	0.59	0.54	0.53	0.63	0.57	0.61	0.56
Correlation coefficient IQR	0.26	0.37	0.37	0.35	0.25	0.26	0.36	0.37
Mean factor contribution	0.14	0.10	0.10	0.08	0.11	0.12	0.13	0.07
Fraction of shared neurons	0.09	0.06	0.19	0.15	0.12	0.15	0.17	0.15
Mean increase in correlation (%)	44.02	30.16	30.17	34.76	32.70	35.88	41.56	23.41
Mean drive ratio	-0.12	0.08	0.09	0.03	-0.04	-0.09	-0.23	0.19
Mean absolute drive ratio	0.57	0.66	0.56	0.70	0.58	0.60	0.61	0.47
