# Supplementary figures and images for: Model-based decoupling of evoked and spontaneous neural activity in calcium imaging data
Source: PLoS Comput Biol. 2020 Nov 30;16(11):e1008330. doi: 10.1371/journal.pcbi.1008330 (PMC7728401; doi:10.1371/journal.pcbi.1008330)

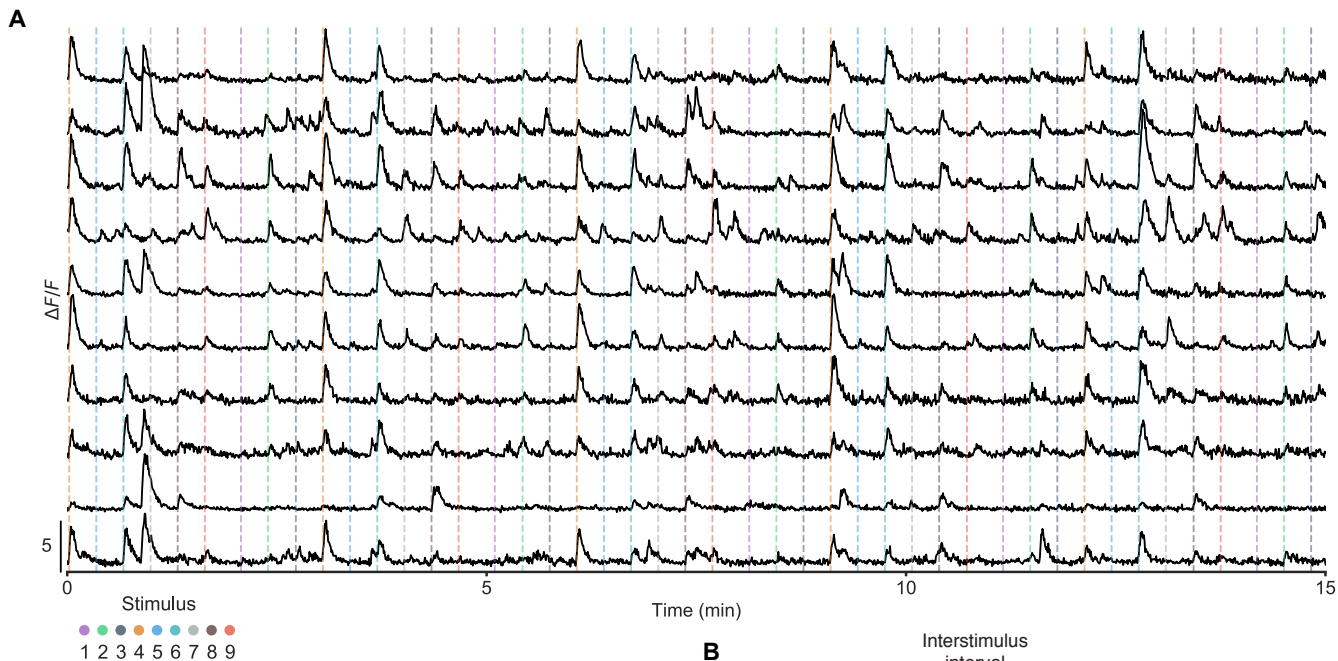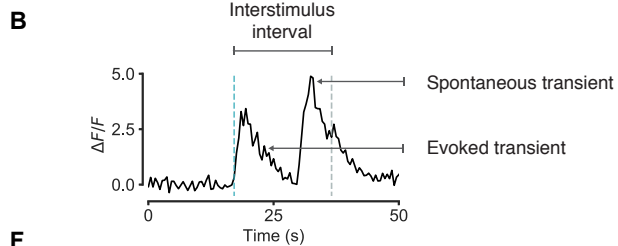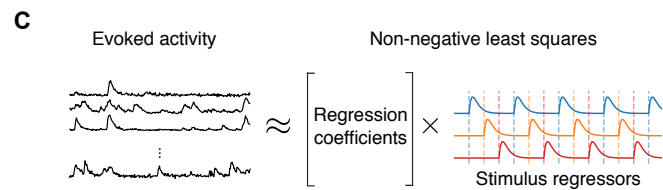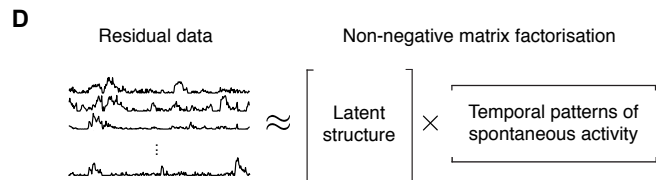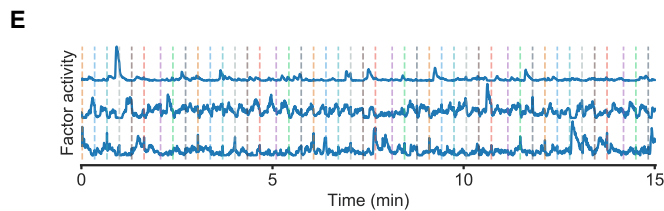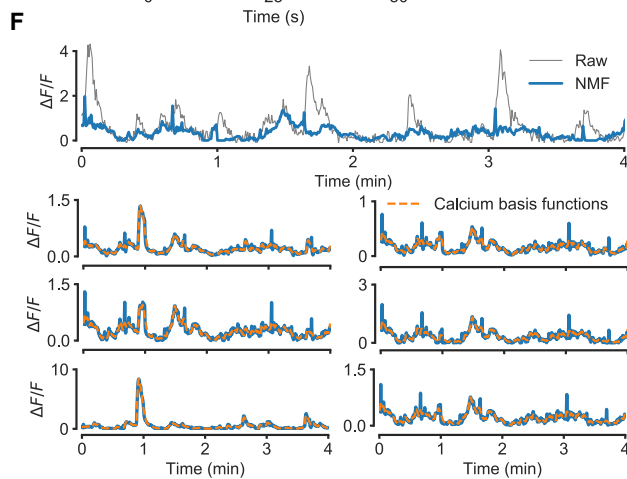

Supplement: S1 Fig — (A) Fluorescence traces from 10 example neurons. Dashed vertical lines indicate stimulus onset; colour represents azimuth angle of presented stimulus. (B) Example fluorescence trace segment illustrating that spontaneous calcium transients can occur just before stimulus onset. (C) A simple estimate of the stimulus-driven component of population data can be obtained by multiple regression of fluorescence traces onto stimulus regressors using non-negative least squares. (D) After estimating the stimulus-driven component, low dimensional structure in the residual data can be estimated using non-negative matrix factorisation. (E) Patterns of SA shared between groups of neurons found via NMF. For consistency with later results, we here applied NMF with three latent factors. Each row corresponds to the activity of one factor. (F) Top: component of the raw fluorescence trace (black) considered to be SA by the residual NMF approach (blue). NMF often produces estimates with erratic and sudden changes in calcium levels that fail to respect the stereotypical structure of calcium activity. Bottom: additional examples of shared SA estimated from the residuals using NMF (blue). For comparison, the same estimates are shown when expressed in a basis of calcium impulse response functions located at each time point (orange, Methods). Deviations from the orange curve demonstrate atypical calcium behaviour. Samples were selected for illustration from among the 10 neurons best explained by the residual NMF approach. (PDF) [file pcbi.1008330.s006.pdf]

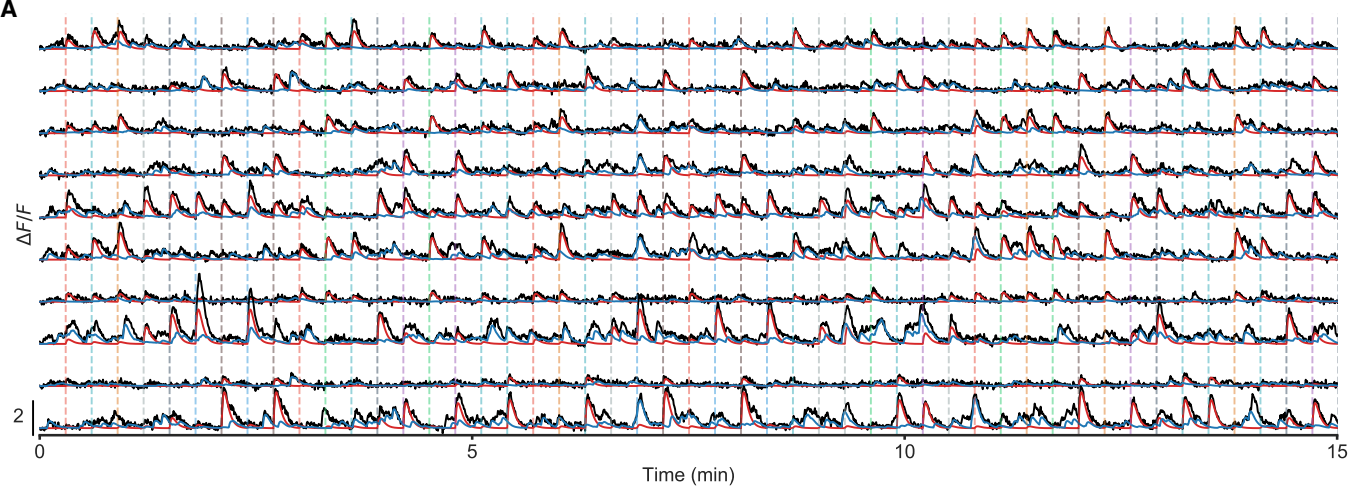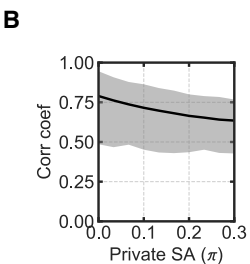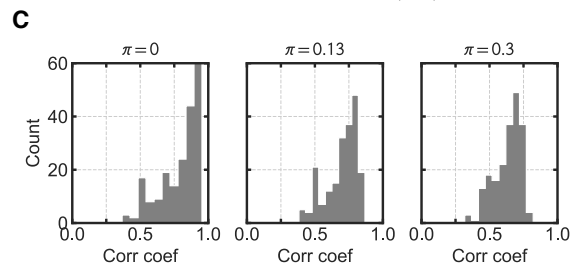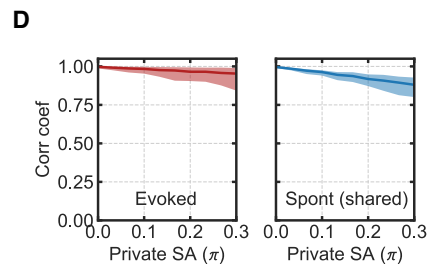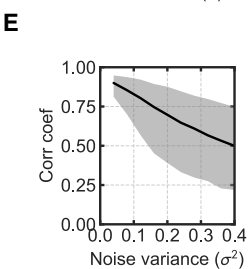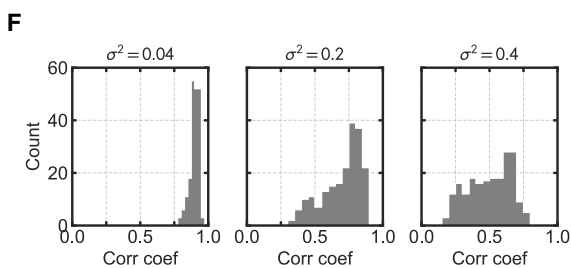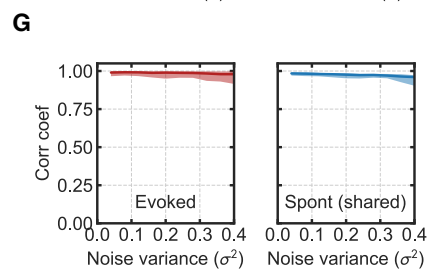

Supplement: S2 Fig — To validate performance we fit the model to simulated data (see Methods). The two primary constraints on model performance are (i) the rate π of private spontaneous events, and (ii) the variance σ2 of the imaging noise. We systematically varied these two parameters and observed the ability of the model to recover the underlying evoked and spontaneous components. Parameters used in the simulations are given in S1 Table. (A) Ten randomly chosen neurons from an example simulation with π = 0.05 and σ2 = 0.1. Black traces show simulated raw fluorescence data. The true composition of the fluorescence trace is given in red (EA) and blue (shared SA). (B) The correlation coefficient between the raw fluorescence trace and model reconstruction decreases as the rate of private spontaneous events increases. (C) Histograms of correlation coefficients for three example values of π. (D), While the correlation coefficient decreases with π, recovery of the evoked (left) and spontaneous (right) fluorescence components remains highly accurate. (E)—(G) Same as (B)—(D) but with varying noise variances σ2. High noise variances limit the correlation between the raw (noisy) fluorescence trace and (noiseless) model reconstruction, but recovery of the evoked and spontaneous components is still very robust. All shaded regions represent 95th percentiles. (PDF) [file pcbi.1008330.s007.pdf]

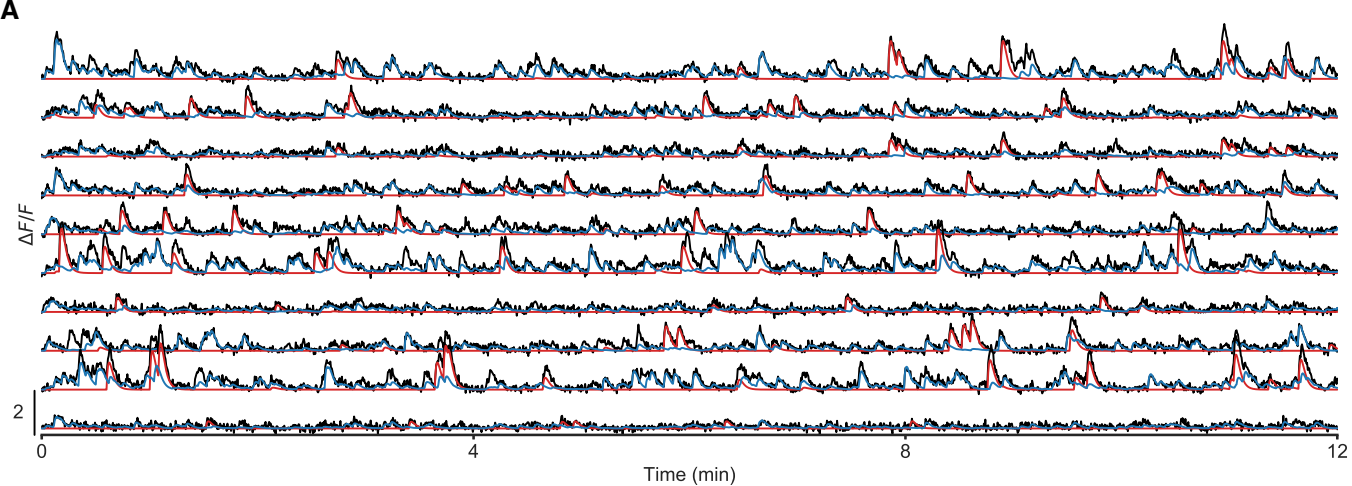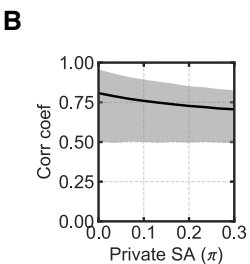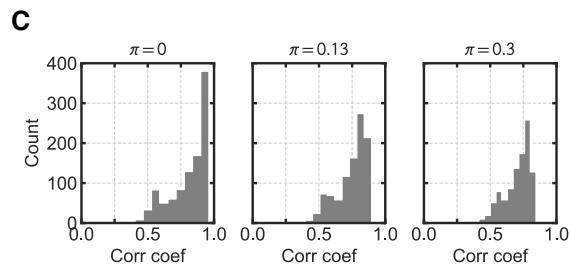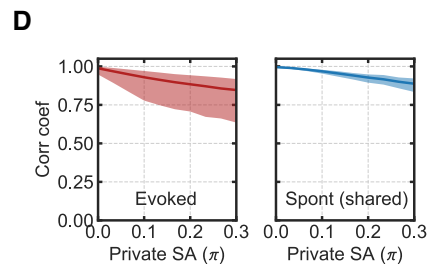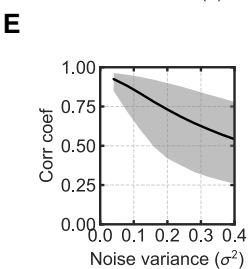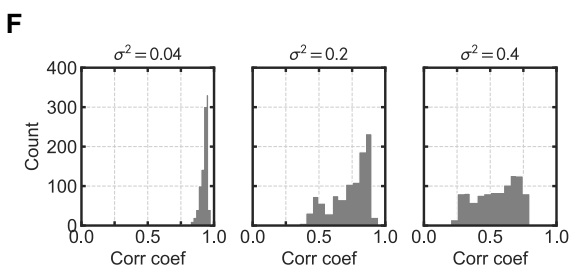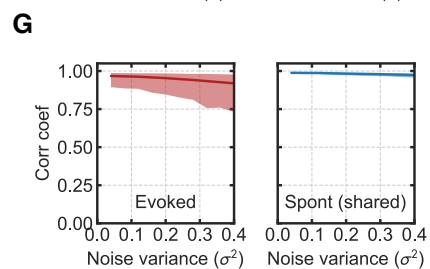

Supplement: S3 Fig — Parameters used in the simulations are given in S2 Table. (A) Ten randomly chosen neurons from an example simulation with π = 0.05 and σ2 = 0.1. Black traces show simulated raw fluorescence data. The true composition of the fluorescence trace is given in red (EA) and blue (shared SA). (B) The correlation coefficient between the raw fluorescence trace and model reconstruction decreases as the rate of private spontaneous events increases. (C) Histograms of correlation coefficients for three example values of π. (D) While the correlation coefficient decreases with π, recovery of the evoked (left) and spontaneous (right) fluorescence components remains highly accurate. (E)—(G) Same as (B)—(D) but with varying noise variances σ2. All shaded regions represent 95th percentiles. (PDF) [file pcbi.1008330.s008.pdf]

**A**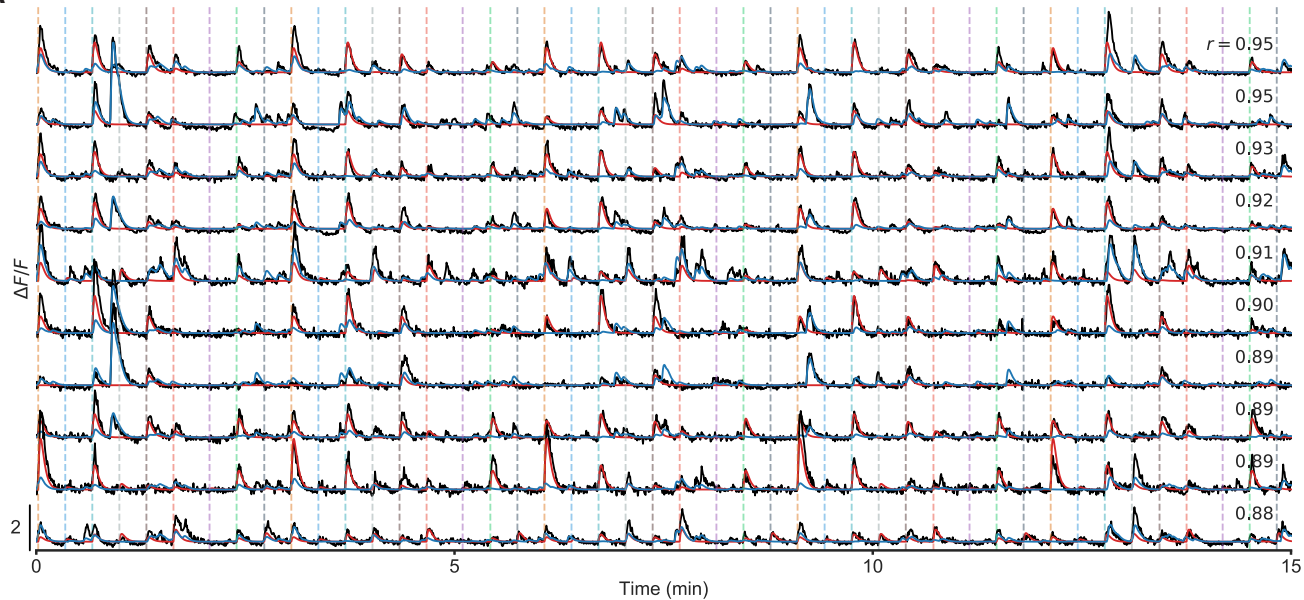**B**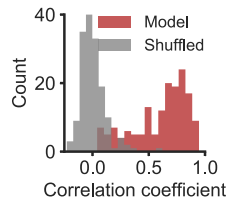**C**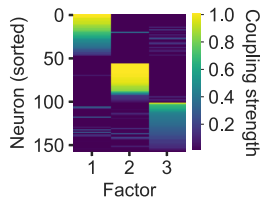**D**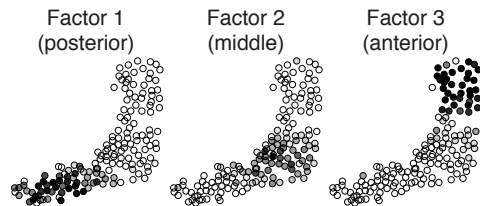**E**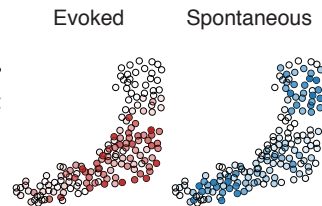

Supplement: S4 Fig — (A) Results of fitting CILVA and decoupling EA (red) and shared SA (blue) in an experimental recording with CaImAn preprocessing (cf. Fig 3A). Inset numbers denote the Pearson correlation coefficient between raw fluorescence trace and model fit. The 10 neurons with the highest correlations between data and model fit are shown. (B) Distribution of correlation coefficients between data and model fits (cf. Fig 3B). Shuffled data obtained by cyclically permuting each trace by a random offset while preserving its temporal structure. (C) Estimated factor coupling matrix shows that latent factors target distinct, non-overlapping sets of neurons. (D) Spatial organisation of latent factors underlying SA (cf. Fig 4G). The three non-overlapping factors are spatially localised and tile the imaging plane. (E) Spatial organisation of the evoked and spontaneous variance components (cf. Fig 4H and 4I). Cell opacity is proportional to the fraction of variance attributable to EA or SA for the given neuron. (PDF) [file pcbi.1008330.s009.pdf]

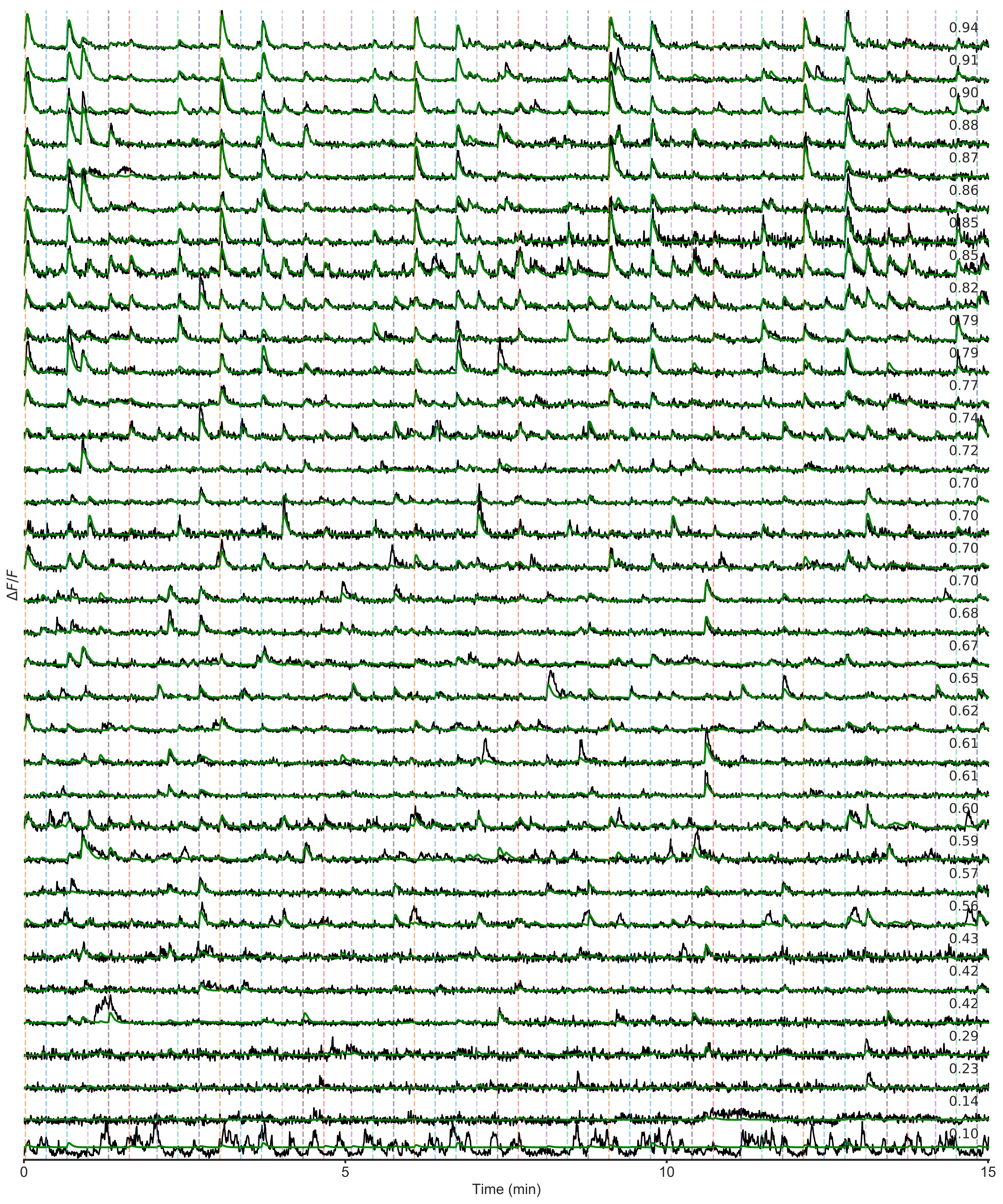

Supplement: S5 Fig — Example fluorescence traces (black) and corresponding model fits (green). Dashed vertical lines indicate stimulus onset times. Inset numbers denote Pearson correlation coefficient between raw trace and model fit. Sampled neurons are sorted by correlation. Poor fits can result from neurons that show inconsistent responses (or no responses) to presented stimuli or neurons dominated by private SA (and therefore that cannot be assigned to a latent factor). Another potential reason the model would fit poorly is segmentation errors when identifying neurons. However, manual inspection of the raw data suggested that this was not the case for the neurons shown here. (PDF) [file pcbi.1008330.s010.pdf]

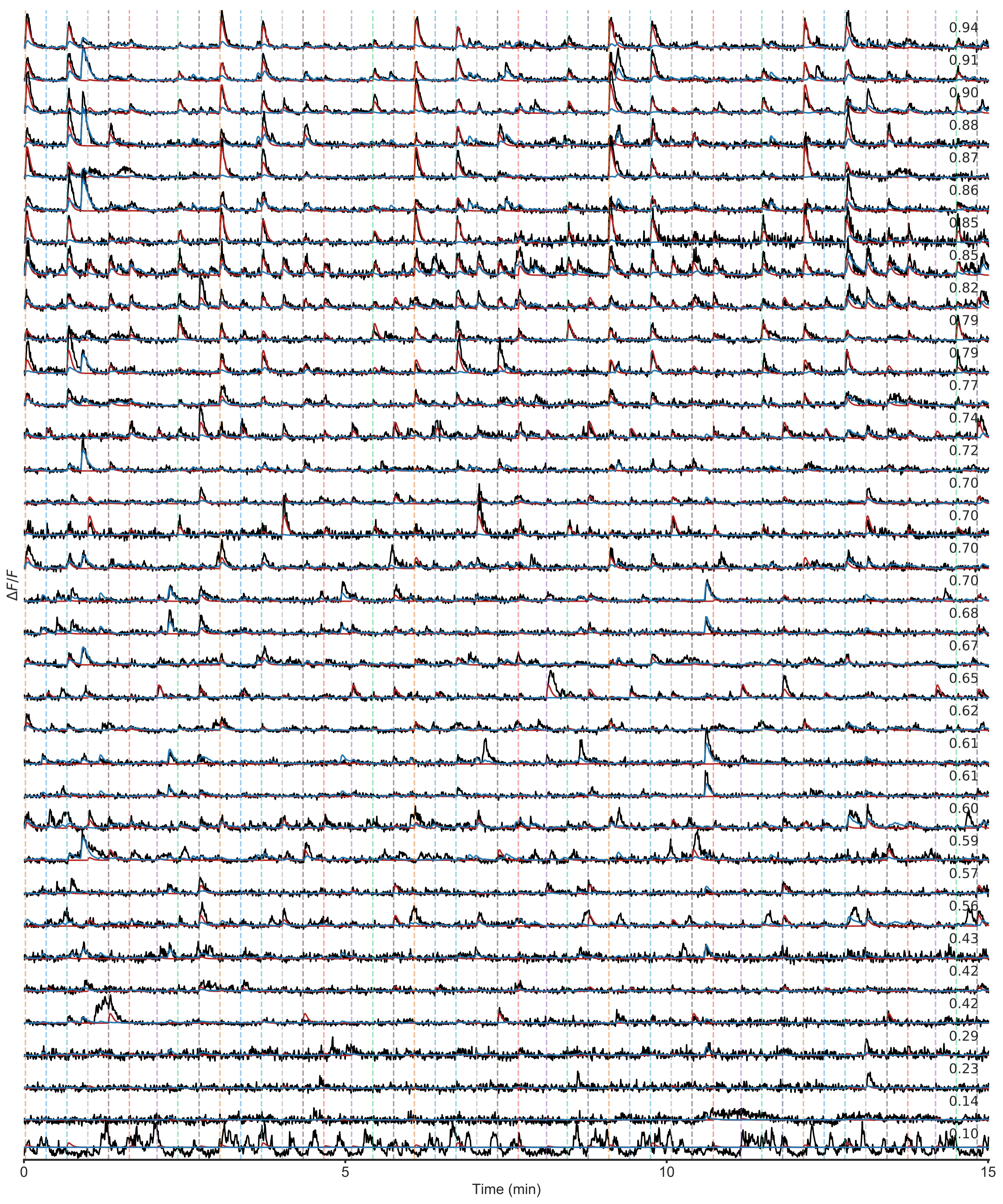

Supplement: S6 Fig — (PDF) [file pcbi.1008330.s011.pdf]

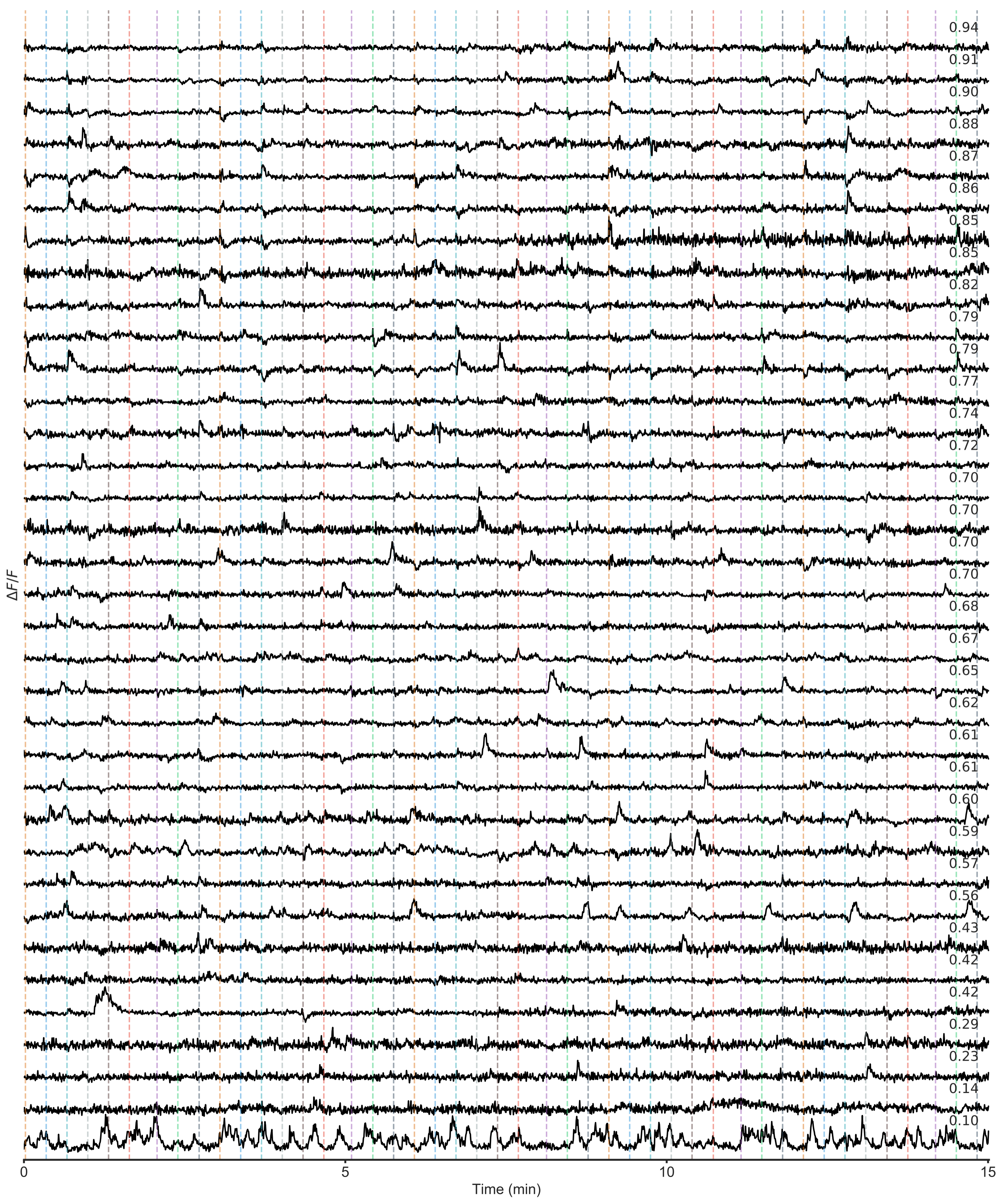

Supplement: S7 Fig — Residual data obtained by subtracting model fit from the raw data (i.e. fn-f^n). Inset numbers denote the correlation coefficients from the model fits in S5 Fig. Ideal residuals appear as independent and identically distributed samples from a Gaussian noise distribution. Systematic deviations from Gaussian noise reflect calcium transients not captured by the model, and contribute to measurements of private variability. (PDF) [file pcbi.1008330.s012.pdf]

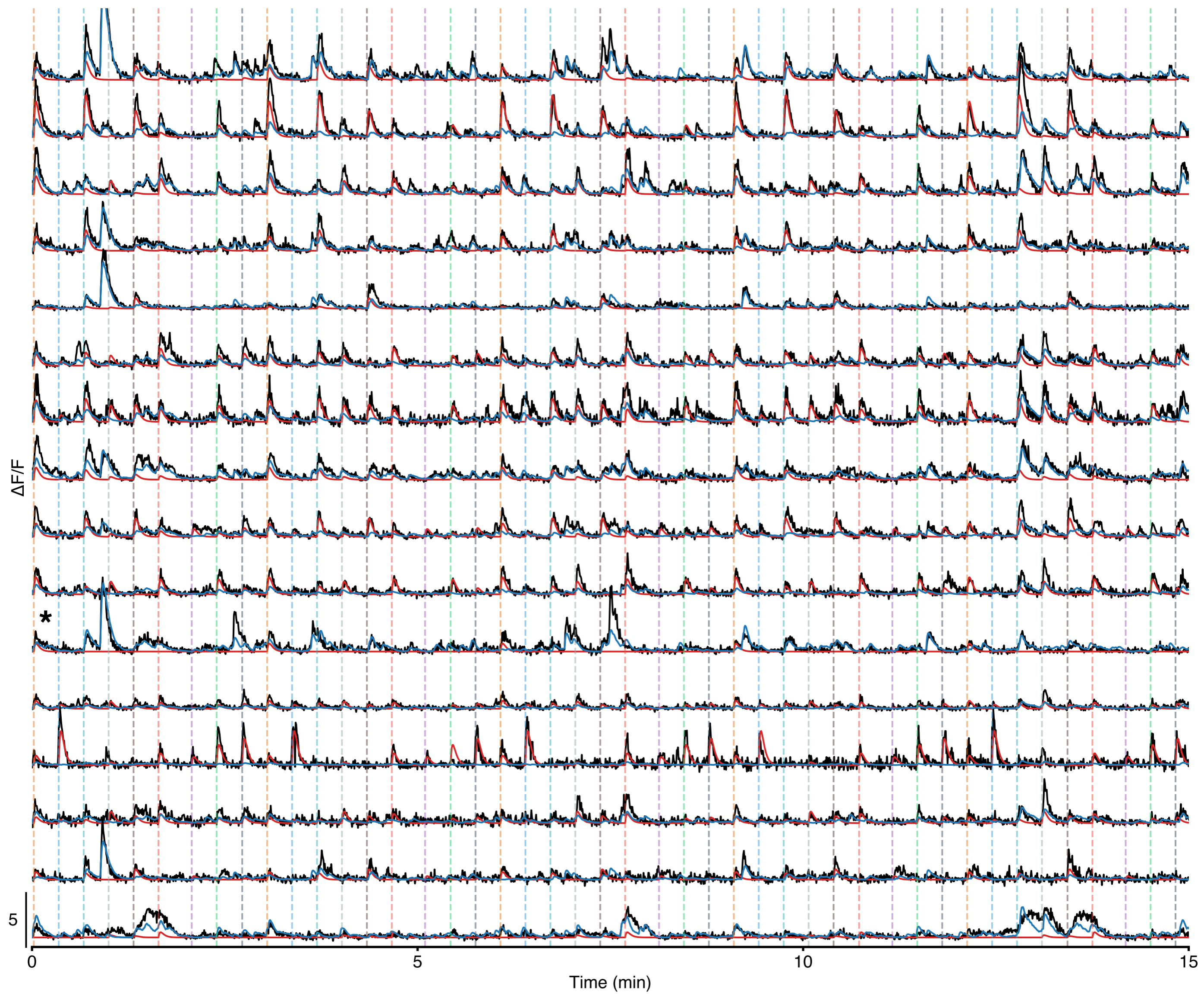

Supplement: S8 Fig — Neurons ordered the same as Fig 3H, with the neuron marked by an asterisk (11th trace) corresponding to the similarly marked neuron in Fig 3H. (PDF) [file pcbi.1008330.s013.pdf]

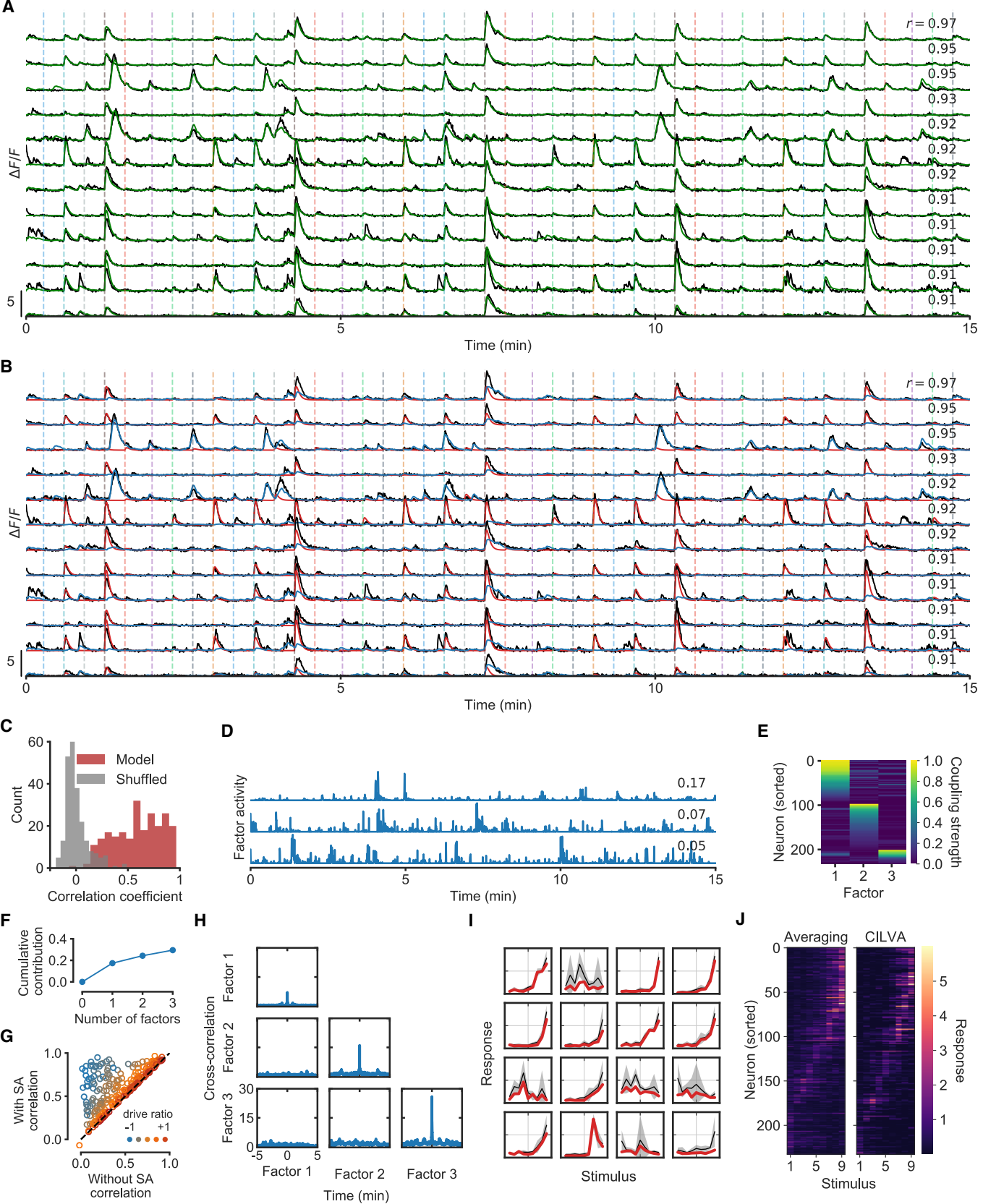

Supplement: S9 Fig — (A) Example fluorescence traces (black) and model fits (green) for the twelve best fitting neurons. Inset numbers denote the Pearson correlation coefficient between raw trace and model fit. (B) Application of the statistical model to decouple EA (red) and shared SA (blue). (C) Distribution of correlation coefficients between data and model fits. Shuffled data (gray) obtained by cyclically permuting each model fit by a random offset while preserving its temporal structure. (D) Inferred latent factor timeseries. Inset numbers denote the factor contribution indices. (E) Factor coupling matrix. (F) Cumulative factor contribution indices for 0-3 latent factors. (G) Correlation coefficient between raw fluorescence trace and model fit with and without incorporation of SA. Neurons with strongly negative drive ratios show marked improvement in quality of model fit. (H) Cross-correlograms show little interaction between latent factors. (I) Example stimulus filters (red). Tuning curves obtained by averaging fluorescence levels over a small window following stimulus presentation provided for comparison (gray). Shaded error bars represent one standard deviation. (J) Retinotopic maps obtained by averaging (left) and by fitting CILVA (right). (PDF) [file pcbi.1008330.s014.pdf]

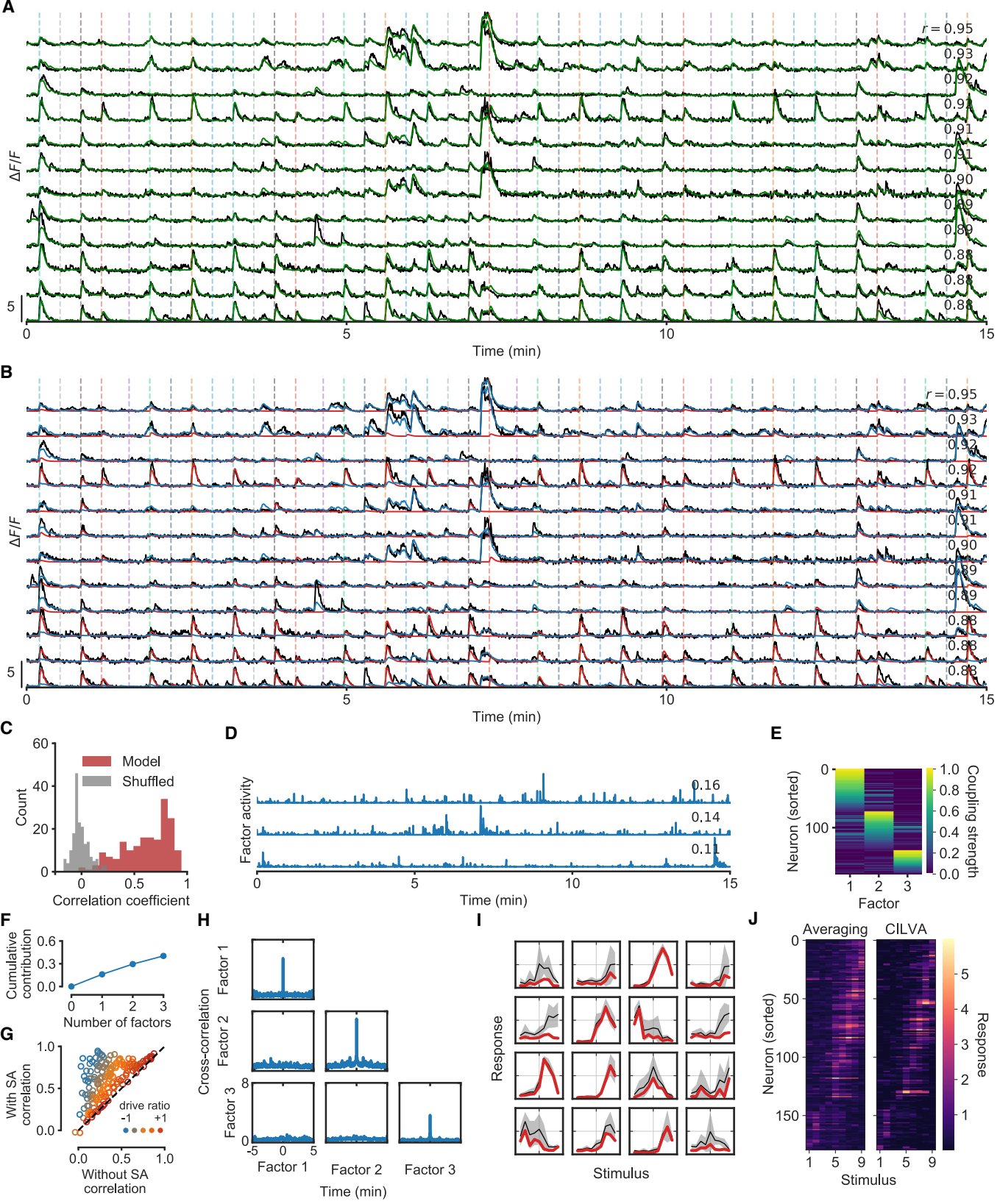

Supplement: S10 Fig — (A) Example fluorescence traces (black) and model fits (green) for the twelve best fitting neurons. Inset numbers denote the Pearson correlation coefficient between raw trace and model fit. (B) Application of the statistical model to decouple EA (red) and shared SA (blue). (C) Distribution of correlation coefficients between data and model fits. Shuffled data (gray) obtained by cyclically permuting each model fit by a random offset while preserving its temporal structure. (D) Inferred latent factor timeseries. Inset numbers denote the factor contribution indices. (E) Factor coupling matrix. (F) Cumulative factor contribution indices for 0-3 latent factors. (G) Correlation coefficient between raw fluorescence trace and model fit with and without incorporation of SA. Neurons with strongly negative drive ratios show marked improvement in quality of model fit. (H) Cross-correlograms show little interaction between latent factors. (I) Example stimulus filters (red). Tuning curves obtained by averaging fluorescence levels over a small window following stimulus presentation provided for comparison (gray). Shaded error bars represent one standard deviation. (J) Retinotopic maps obtained by averaging (left) and by fitting CILVA (right). (PDF) [file pcbi.1008330.s015.pdf]

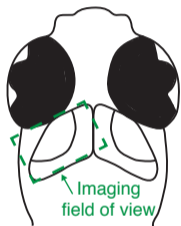

Zebrafish x8

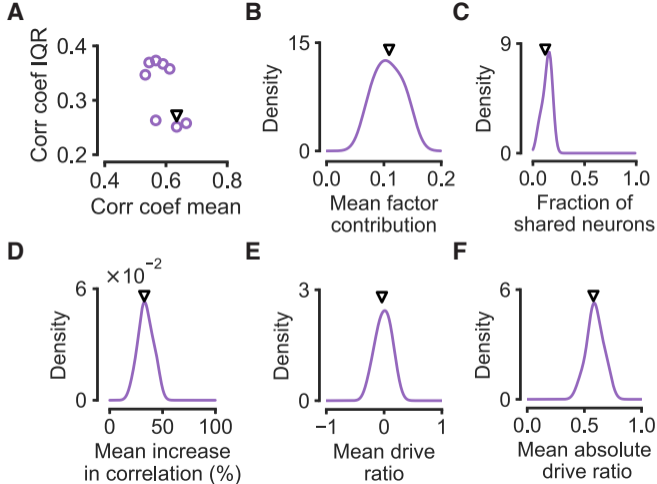

Supplement: S11 Fig — Black triangles point to the example fish from the main text. (A) Mean and interquartile range (IQR) of correlation coefficient distributions for n = 8 larvae. (B) Distribution of factor contribution indices. For model fits with 3 latent sources of SA, each factor has a contribution index of ∼ 0.1. (C) Distribution of fraction of neurons ‘shared’ between multiple factors. Neurons were considered shared if they were coupled to more than one factor with coupling strengths exceeding a threshold of 25% of the maximum coupling strength for that factor. (D) Mean improvement in correlation coefficients with incorporation of latent sources of SA. (E) Distribution of mean drive ratios across the population of larvae, centered at −0.01, suggesting that SA and EA are largely balanced within individual fish. (F) The mean absolute values of the drive ratio are greater than 0, showing that individual neurons tend to be biased towards either EA or SA. Histograms in panels B—F obtained by non-parametric density estimation with Gaussian kernels. Raw data points used for histograms given in S3 Table. (PDF) [file pcbi.1008330.s016.pdf]

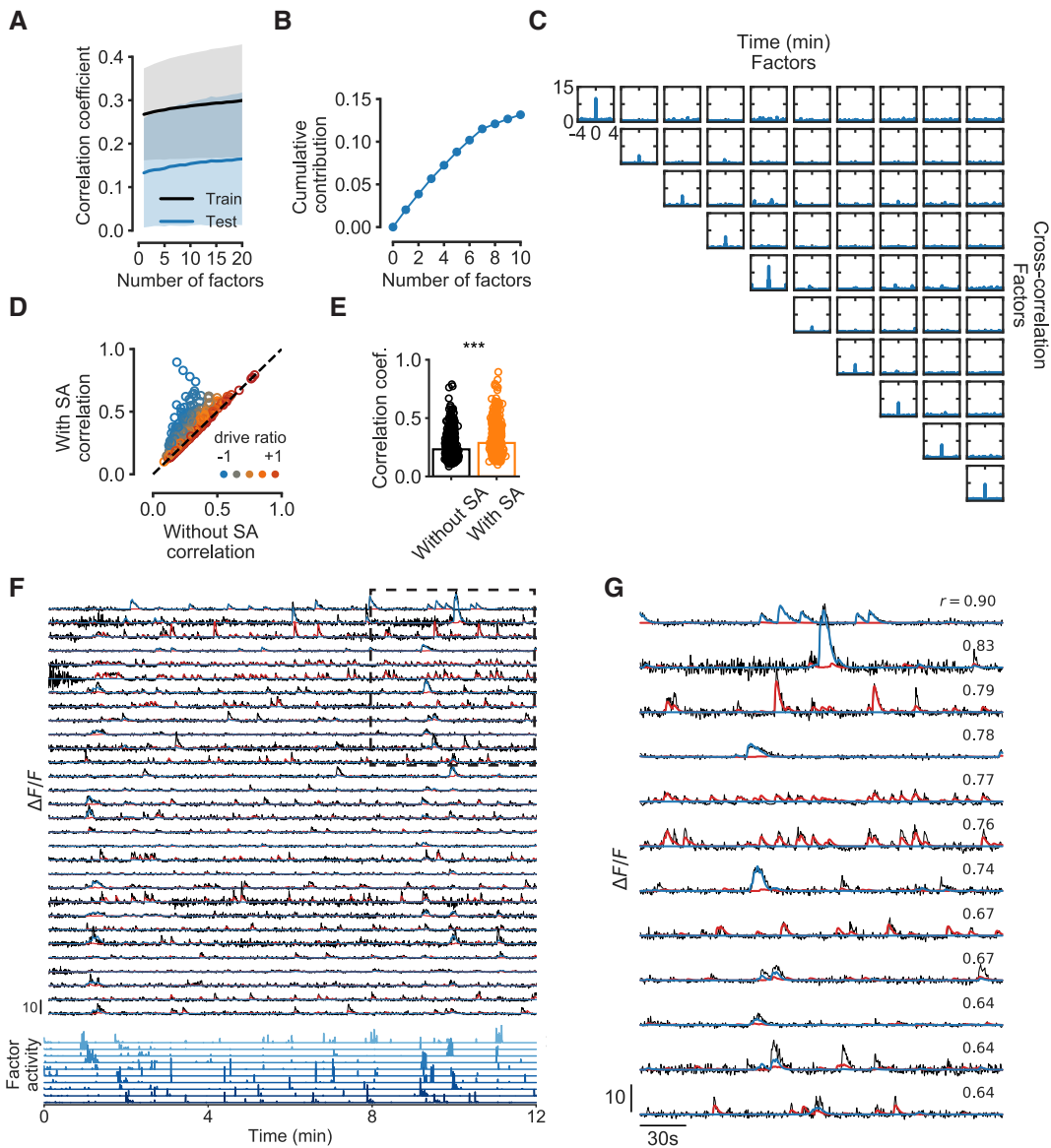

Supplement: S12 Fig — (A) We fit the model with 10 latent factors. While the contribution indices for the factors gradually diminished (panel D), varying the number of factors from 1 to 20 did not identify a point at which the overall quality of fit failed to increase, including in held-out test data. (B) Cross-correlograms between latent factor timeseries indicate factors underlying SA are mutually independent. (C) Cumulative contribution of factors to quality of model fit. (D) Correlation coefficients between raw fluorescence trace and model fit with and without the SA component. Neurons with negative drive ratios (blue circles) demonstrate substantial improvement in the quality of model fit when incorporating SA. (E) Improvement in the quality of model fit when incorporating the SA component is statistically significant (p < 0.001, Wilcoxon signed-rank test). (F) Example decoupling of EA and SA for the 30 best fit neurons (top) and underlying latent factor timeseries (bottom). (G) Close-up of model fit from neurons in dashed region in panel G. Inset numbers denote Pearson correlation between raw data and full model fit. (PDF) [file pcbi.1008330.s017.pdf]
