# Supplementary material for: Model-based decoupling of evoked and spontaneous neural activity in calcium imaging data
Source: PLoS Comput Biol. 2020 Nov 30;16(11):e1008330. doi: 10.1371/journal.pcbi.1008330 (PMC7728401; doi:10.1371/journal.pcbi.1008330)
Supplement: Attachment — Submitted filename: response.pdf [file pcbi.1008330.s018.pdf]

Model-based decoupling of evoked and spontaneous neural activity in calcium imaging data

Response to reviewers

Marcus A. Triplett, Zac Pujic, Biao Sun, Lilach Avitan, and Geoffrey J. Goodhill

September 6, 2020

We thank the reviewers for their positive evaluation of our manuscript and their useful comments. We respond to each of these below.

Response to Reviewer 1

1. *What causes neurons to have a poor model fit, eg the neurons at the left side of the red distribution in Fig 3B? I am curious if the authors have inspected these neurons to see if they might be poorly sampled in the imaging data, improperly segmented, and/or otherwise 'bad' in a way that might explain the poor performance of the model. My goal by asking this is to improve the work yet further by attempting to understand why it does not work when the model fit is poor. The authors mention in a few places that the neurons that are not fit well may be generally quiescent, unresponsive to sensory stimuli, and/or driven by some spontaneous activity not shared with other neurons recorded. But perhaps the issue is not the model but the data?*

This is a good point. In response we manually inspected some of the worst-fitting cells in the raw, unsegmented recording, but found that they almost all appeared to be properly segmented. However, we do agree that poor segmentation could be a source of poor fit, and we now mention this possibility in the caption of Figure S5.

2. *Is it possible to show the raw traces for a neuron with an inferred tuning that is very different from the one obtained by averaging as showing in H? The raw traces in 3A are very useful for understanding how the CILVA approach helps. And the tuning curves shown in H show some substantial differences. Is it possible some of the neurons in 3A happen to be some of the neurons in 3H already? I am curious for example about the neuron in the second from the bottom row and second from the last column in 3H — this is one place where the approach seems to massively beat the standard approach!*

We thank the reviewer for raising this point, and agree that these are useful traces to see. We have now included an additional supplementary figure that shows every trace corresponding to the tuning curves in Figure 3H (see Figure S8 in revised manuscript). The particular neuron mentioned by the reviewer (11th neuron in Figure 3H, now highlighted with an asterisk) is shown in the supplementary figure (11th fluorescence trace) to be almost entirely spontaneous activity, with only a small calcium transient consistently evoked by the stimulus colour-coded in orange. We agree with the reviewer that this is an excellent example of the utility of the model, and now reference this example in the main text (line 222).

3. *Line 159-161: "we encouraged sparsity by placing a non-negative prior on the latent factors with high density near zero, and used a simple model selection procedure to estimate the sparsity penalty" I'm*

curious why the authors did not use lasso or ridge to enforce sparsity. This also effects the model fitting possibilities (from Line 533).

In CILVA the latent variables are non-negative. Thus, maximising the log-likelihood with a lasso regulariser (which penalises the L1 norm of the latents) is mathematically equivalent to maximising the log-likelihood with an exponential prior on the latent variables. We have expanded on this point in the Methods (line 507).

4. *Fig 1B bottom left stimulus colour code seems redundant as it provides no extra information given the order of stimuli can be inferred from the sequence of coloured dashed vertical lines.*

The colours (chosen to be mutually discernable) correspond to angles in space in the order given by the key, and the dashed lines show the stimulus timing. Our intention is simply to clearly outline the set of colours associated with stimuli.

5. *Lambda is referred to as "rate of calcium influx" in line 143, "underlying intensity of neural activity" in Fig 2A legend, and finally "Intensity functions" in Fig 2B legend. It is bold in Fig 2 legend but not Fig 2 itself or line 143. Please describe and use consistently.*

We apologise for not being consistent with this. We originally referred to lambda as an intensity to draw an analogy with GLMs for neural spike train data. We now consistently refer to this as the rate of calcium influx. We follow a convention where bold-face characters refer to vectors/matrices (as in e.g., Bishop 2006).

- Line 133: *"the calcium dynamics are assumed to be highly stereotyped and are defined by the convolution of a calcium impulse response kernel k with an activity intensity function"* is now *"the calcium dynamics are assumed to be highly stereotyped and are defined by the convolution of a GCaMP impulse response kernel k with a vector of calcium influxes (analogous to an activity intensity function)".*
- Figure 2A caption: *"These two sources are combined additively to define the underlying intensity of neural activity (λ_n), before being convolved with a calcium kernel"* is now *"These two sources are combined additively to define the underlying rate of calcium influx (λ_n), before being convolved with a GCaMP kernel."*
- Figure 2B caption: *"Intensity functions λ_n encoding stimuli and shared SA are convolved with a calcium impulse response kernel k to generate calcium levels"* is now *"The intensity of calcium influx λ_n encoding stimuli and shared SA are convolved with a GCaMP kernel k to generate observed calcium levels."*

6. *Line 238 "well-describes" odd phrasing.*

We have now changed this to "is a good description of" (line 239).

7. *"Table S3: Raw data points for the histograms in Figure 5." Does not appear to refer to Figure 5.*

We now correctly refer to Figure S10.

8. *It seems odds to present the grand average data in S10 rather than a main figure? My opinion is not strong but if other reviewers make a similar remark perhaps moving it to a main figure would be appropriate.*

Our intention with this figure is to provide assurance that the outcome of applying CILVA was not a fluke, and that the subsequent statistical properties of the data was consistent across a set of different animals. We have now clarified this in the main text (line 267). We see this result as mainly 'housekeeping', and hence placed it as a supplementary figure.

Reviewer 2

1. *Dynamics of the latent factors. In the traces in Fig3A it looks like several spontaneous "bumps" are evoked by the stimulus. As far as I can tell, this tendency is not quantified except for the size of the orange 'covariance' bars in Fig4A. Somehow looking at 4A it seems like the covariance is marginal, but then looking at 3A it's easy to see 'by eye' the evoked factor transients. It should be quite straightforward for the authors to quantify these evoked factor transients.*

More generally, what are the implications for the model of a situation where spontaneous events are 'triggered' by the stimulus? In the cortex, phenomena like this have been well characterised in the dynamics of e.g., up-states (Luczak & Harris, Hasenstaub & McCormick, etc). Up-states can and do occur spontaneously, but they are easily evoked. When the interval of stimulus presentation is regular (as in the current study?), the timing of spontaneous population events has even been shown to track (not presented) stimuli! (Li, Jingcheng, et al. "Primary auditory cortex is required for anticipatory motor response." Cerebral Cortex 27.6 (2017): 3254-3271).

Conceivably, every (or a large majority of) spike comes from these population events and the stimulus simply triggers them sometimes. Is this scenario describable by the model? Somehow the additive nature of the interaction between stimulus and factors does not lend itself easily to describe this scenario in my mind, but maybe I'm missing something? Could the authors elaborate on this point?

This is an interesting point and we have now added the following text to the Discussion (lines 326-336): "The interaction between EA and SA could also affect the underlying dynamics of the neural activity. This could occur if, e.g., the presentation of a stimulus engages recurrent circuits that trigger the activation of a latent factor. In the case of our data in Figure 4H and I, the distinct spatial organisation of the evoked and spontaneous variance components indicate that this triggering effect is not likely to be a predominant source of variation (although there is some overlap in these two components in the middle tectum). Although this kind of "triggering" interaction is not something the model attempts to explicitly describe, CILVA can potentially account for this effect depending on how the triggering occurs. If the triggering of a factor always occurs with stimulus presentation, then this will be incorporated into the receptive field component. If the triggering of a factor occurs only occasionally and with a sufficiently large amplitude, then this will be associated with a latent factor instead."

2. *It would be useful to mention explicitly the part of the variability which is private when describing the model. Although private variability is mentioned several times (pages 12, 20,21...) I could not find a mention to it in the description of the formulas (only for simulated data).*

We thank the reviewer for raising this point. Private variability is not an explicit component of the model. Rather, this is indirectly estimated by subtracting the model-estimated shared variance from the total variance. We have now expanded the methods to make this calculation explicit (line 542).

3. *Positivity constraints. Given that the authors are modelling DeltaF/F, which can be negative, perhaps a comment can e made on the virtues of the positivity constraint on the factors?*

While the $\Delta F/F$ can be negative, this is considered to be a consequence of the imaging noise rather than a negative concentration of bound GCaMP. This is, for example, why calcium imaging preprocess-

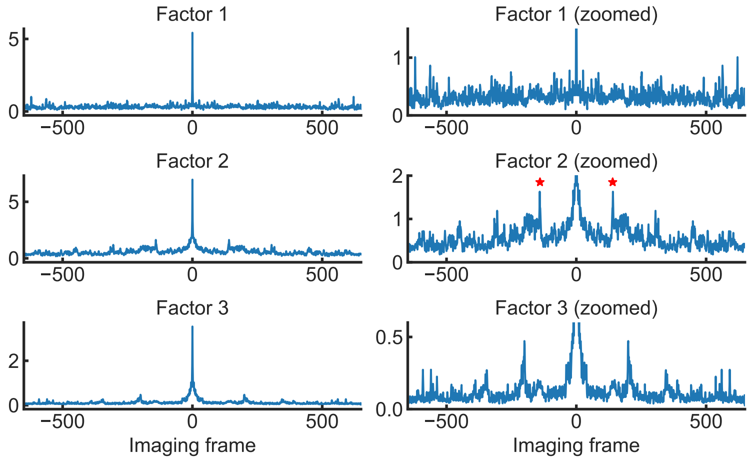

Figure 1: Autocorrelation of the latent factors. Each row corresponds to one factor. The left column includes the peak at zero. The right column highlights any secondary peaks. The timescale of the secondary peaks do not correlate with the timing of the stimuli.

ing methods are based on non-negative deconvolution and non-negative matrix factorisation. We have expanded the Methods to address this point (line 509).

4. *Autocorrelation of the factors. I could not understand the argument given for justifying the lack of autocorrelation of the factors. While certainly simplicity is an argument (and a good one if the model generally works well), the authors rather point to a possible ambiguity between the time-scale of the autocorrelation of the factors and that of the fluorescence due to the Ca-dynamics. I must have missed something, because the Ca-dynamics is already explicitly built into the model through the kernel k. Looking at the autocorrelations of the factors in, e.g, Fig3G, it seems like sometimes there are slow dynamics (for this fish in factor 3). This is not to say that assuming no autocorrelation is a negative feature per se. I just didn't understand if the motivation was simplicity or something else.*

We thank the reviewer for raising this interesting point. Recent work from Wei et al. [1] has shown that, among a number of potential models for calcium imaging data, a "spike-and-slab" approach most effectively captures the statistical properties of the data. Under a spike-and-slab model, calcium influxes are occasionally positive but a majority of the time are exactly zero, and therefore sparse. An exponential prior that specifically omits factor autocorrelation therefore allows us to compromise between this optimal sparsity, model simplicity, and computational tractability. Indeed, among distributions on the non-negative real line with a given mean, the exponential prior has maximum entropy, and therefore minimises the amount of prior information required by the user. We have extended the model description in the Methods to expand on this point (line 523).

5. *Fig3G and equivalent panels in other figures. The y-lim in these plots (set to include the peak at zero) is unfortunate, as it prevents the reader from assessing the temporal structure of the signals. In some cases like factor 2 if FigS9H and others, there even seems to be some oscillatory structure (what is the period of this oscillation? How does it compare to the inter-stimulus interval?).*

We thank the reviewer for raising this point. In our inspection of the factor autocorrelation across our pool of zebrafish we found no noteworthy structure. For example, in the attached Figure 1 we show the autocorrelation in more detail for Figure S9, the example highlighted by the reviewer. For factor 2, the secondary peaks occur 140 frames from the peak at 0 (Figure 1, red stars). However, 140 frames

corresponds to ∼65s given our imaging rate, whereas stimuli are presented for 1s with 19s inter-stimulus intervals (9 stimuli per trial), followed by an inter-trial interval of 25s. The presentation of any particular stimulus therefore has a period of 185s. Thus this secondary peak in autocorrelation has no obvious connection to the stimulus protocol, as one could expect if the temporal structure of the spontaneous activity were largely independent of the stimulus-driven activity. We now comment on this in the caption of Figure 3G.

6. *Topography of the dynamics. The results in Fig4G-I are nice, but I didn't understand the sorting of neurons in 4A. The legend says neurons are sorted by A-P coordinate, but 4A is not nearly as ordered and structured as 4G. Given that the shape of the analysed activity is effectively 1D (although slightly curved), can neurons in 4A be sorted according to this 1D axis? If so, we would see the factor loadings in 4A bottom nicely ordered.*

We thank the reviewer for this suggestion. We have now sorted Figure 4A using the same ordering as Figure 3E, which now more clearly reveals the composition of evoked and spontaneous variance within each factor.

7. *Fig4C. Again, this format seems not ideal for the info that this plot is supposed to convey (maybe a scatter plot like 4C?). Looking at 4C, the difference in covariance looks marginal. It seems to me like it should be possible to test the hypothesis that the covariance in the sample is significantly larger than that of a null model for each neutron.*

We have now changed this to a scatter plot and agree that it improves the visualisation. Our objective with this figure is to report whether individual neurons show significant covariances between their evoked and spontaneous activity components rather than the entire population. Indeed, as we show in subsequent figures, many neurons are dominated by only one of either evoked or spontaneous activity, and we would not expect a significant difference in this case.

8. *Fig 4F. I think a violin plot would make this figure nicer and more transparent.*

We have now changed this to a violin plot, which we agree is a nicer representation of these data.

References

[1] Wei et al. (2020). A zero-inflated gamma model for post-deconvolved calcium imaging traces. *Neurons, Behavior, Data Analysis, and Theory.* doi: 10.1101/637652.
